# Prior Stroke in PFO Patients Is Associated With Both PFO-Related and -Unrelated Factors

**DOI:** 10.3389/fneur.2020.00503

**Published:** 2020-06-04

**Authors:** Timo Kahles, Patrik Michel, Alexander Hapfelmeier, Franz R. Eberli, Marialuisa Zedde, Vincent Thijs, Markus Kraemer, Stefan T. Engelter, Joaquin Serena, Christian Weimar, Achim Mallmann, Andreas Luft, Dimitri Hemelsoet, David E. Thaler, Andreas Müller-Eichelberg, Adinda De Pauw, Roman Sztajzel, Carmel Armon, David M. Kent, Bernhard Meier, Heinrich P. Mattle, Urs Fischer, Marcel Arnold, Marie-Luise Mono, Krassen Nedeltchev, for the International PFO Consortium NCT00859885

**Affiliations:** ^1^Department of Neurology, Cantonal Hospital Aarau, Aarau, Switzerland; ^2^Department of Neurology, University Hospital of Lausanne (CHUV), Lausanne, Switzerland; ^3^Institute of Medical Informatics, Statistics and Epidemiology, School of Medicine, Technical University Munich, München, Germany; ^4^Department of Cardiology, Municipal Hospital Triemli, Zurich, Switzerland; ^5^Department of Neurology, Azienda Unità Sanitaria Locale–IRCCS, Reggio Emilia, Italy; ^6^Department of Neurology, University Hospitals of Leuven, Leuven, Belgium; ^7^Stroke Division, Florey Institute of Neuroscience and Mental Health, University of Melbourne, Melbourne, VIC, Australia; ^8^Department of Neurology, Alfried-Krupp Krankenhaus, Essen, Germany; ^9^Department of Neurology, Heinrich Heine University Duesseldorf, Duesseldorf, Germany; ^10^Department of Neurology, University Hospital of Basel, Basel, Switzerland; ^11^Felix-Platter Hospital, Basel, Switzerland; ^12^Department of Neurology, University Hospital of Girona, Girona, Spain; ^13^Department of Neurology, University Hospital of Essen, Essen, Germany; ^14^Department of Neurology, Klinikum Worms, Worms, Germany; ^15^Department of Neurology, University Hospital of Zurich, Zurich, Switzerland; ^16^Department of Neurology, University Hospital of Ghent, Ghent, Belgium; ^17^Department of Neurology, Tufts Medical Center, Boston, MA, United States; ^18^Ammerland Klinik, Westerstede, Germany; ^19^Department of Neurology, AZ Sint Blasius, Dendermonde, Belgium; ^20^Department of Neurology, University Hospital of Geneva, Geneva, Switzerland; ^21^Department of Neurology, Baystate Health Center, Springfield, MA, United States; ^22^Sackler School of Medicine and Department of Neurology, Yitzchak Shamir Medical Center, Tel Aviv University, Tel Aviv-Yafo, Israel; ^23^Institute for Clinical Research and Health Policy Studies, Tufts Medical Center, Boston, MA, United States; ^24^Department of Cardiology, University Hospital of Bern, Bern, Switzerland; ^25^Department of Neurology, University Hospital of Bern, Bern, Switzerland

**Keywords:** patent foramen ovale, PFO, right-to-left shunt, cryptogenic stroke, prior stroke, risk factor, hypercholesterolemia, International PFO Consortium

## Abstract

**Background and Purpose:** To identify factors associated with prior stroke at presentation in patients with cryptogenic stroke (CS) and patent foramen ovale (PFO).

**Methods:** We studied cross-sectional data from the International PFO Consortium Study (NCT00859885). Patients with first-ever stroke and those with prior stroke at baseline were analyzed for an association with PFO-related (right-to-left shunt at rest, atrial septal aneurysm, deep venous thrombosis, pulmonary embolism, and Valsalva maneuver) and PFO-unrelated factors (age, gender, BMI, hypertension, diabetes mellitus, hypercholesterolemia, smoking, migraine, coronary artery disease, aortic plaque). A multivariable analysis was used to adjust effect estimation for confounding, e.g., owing to the age-dependent definition of study groups in this cross-sectional study design.

**Results:** We identified 635 patients with first-ever and 53 patients with prior stroke. Age, BMI, hypertension, diabetes mellitus, hypercholesterolemia, coronary artery disease, and right-to-left shunt (RLS) at rest were significantly associated with prior stroke. Using a pre-specified multivariable logistic regression model, age (Odds Ratio 1.06), BMI (OR 1.06), hypercholesterolemia (OR 1.90) and RLS at rest (OR 1.88) were strongly associated with prior stroke.Based on these factors, we developed a nomogram to illustrate the strength of the relation of individual factors to prior stroke.

**Conclusion:** In patients with CS and PFO, the likelihood of prior stroke is associated with both, PFO-related and PFO-unrelated factors.

## Introduction

The prevalence of patent foramen ovale (PFO) in the general adult population is 15–35% (1) and its association with cryptogenic stroke (CS) has been clearly established (2, 3). The higher prevalence of PFO in CS of all ages (3, 4) suggests a pathogenic role for PFO, at least in a substantial portion of these patients. Assuming that paradoxical embolism is the predominant pathogenic mechanism for recurrent strokes (5), PFO closure is a logical treatment option. However, recent RCTs comparing percutaneous closure with antithrombotic treatment revealed inconsistent results—some of them in favor of closure (6–9), whereas others without a significant advantage of closure (10–13). Low recurrence rates under both prevention regimens, non-PFO related recurrent stroke mechanisms, crossovers, procedure- and device-related complications as well as suboptimal patient selection—i.e., including some patients with non-PFO-related index strokes—might explain the inconsistency of the results (14–17). Hence, in patients with PFO and CS, the risk of stroke recurrence may be associated with both PFO-related and PFO-unrelated factors.

Previous strokes at presentation have been identified as a risk factor for stroke recurrence in patients with CS and PFO (18).

The aim of this study was to identify PFO-related and -unrelated risk factors associated with prior stroke in CS patients. Furthermore, we developed a nomogram to illustrate the strength of these associations.

## Methods

### Patients

The International PFO Consortium is an ongoing academic trial, where researchers from currently nineteen stroke centers worldwide collaborate (NCT00859885). It was founded in 2008 and collects data of patients with ischemic stroke or TIA and PFO. Emphasis is placed on the evaluation of risk factors, PFO diagnosis, and secondary stroke prevention. It is a multicenter prospective study with a scheduled yearly follow-up. Database is expected to be closed after all patients reach a minimum of three years follow-up in 2021. Ethical approval was obtained from the local ethics committee of the corresponding center if legally required.

Patients older than 18 years with ischemic stroke or TIA ≤ 3 months and proven PFO on transesophageal echocardiography are eligible for the International PFO Consortium Study. There was no upper age limit. The whole International PFO consortium cohort included patients with different stroke etiologies. In the current study we addressed those with an undetermined stroke etiology, i.e., CS. Baseline data comprise demographic data, vascular risk factors, conditions predisposing to paradoxical embolism, previous medication, brain CT or MRI findings, echocardiographic PFO-features, and stroke etiology according to TOAST criteria (19). Annual follow-up visits assess secondary stroke prevention and stroke recurrence. Vascular risk factors include age, gender, arterial hypertension, diabetes mellitus, hypercholesterolemia, smoking, self-reported migraine, coronary artery disease, previous stroke, thrombophilia (factor V Leiden and prothrombin mutation, protein C and S deficiency, AT3 deficiency, and antiphospholipid antibodies). Echocardiographic features include atrial septal aneurysm (ASA) defined as hypermobility of the atrial septum with an excursion of >10 mm from midline, aortic plaques >4 mm thickness, and right-to-left shunt (RLS) at rest or under Valsalva maneuver (VM). Conditions predisposing to paradoxical embolism comprise VM at the time of stroke onset, deep vein thrombosis (DVT), and pulmonary embolism.

From September 2008 through December 2014, the International PFO Consortium enrolled 931 patients with CS and PFO. The present study focused on two patient subgroups: (a) 635 patients with first-ever stroke (i.e., neither radiological nor clinical evidence of prior stroke) and (b) 53 patients with prior stroke (i.e., both clinical and radiological evidence of prior stroke). Patients, who could not unambiguously assigned to the first-ever or the recurrent stroke group on the basis of past medical history and radiological signs, i.e., CS patients with clinical but no radiological evidence of prior stroke or vice versa (*n* = 243) were not included in the present study.

### Statistical Analysis

The distribution of quantitative data is described by mean ± standard deviation. Qualitative data is presented by absolute and relative frequencies. Corresponding hypothesis testing was performed by *t*-Test and the Chi-squared test or Fisher's exact test, as appropriate.

Missing values were imputed using a Random Forests model to account for possible interactions and high-dimensional relations of the data (20). Associations with prior stroke were estimated by Odds Ratios, with 95% confidence intervals, using univariate and multivariable logistic regression models. Any model contained age as an independent variable to adjust for confounding by the time-dependent stroke risk. Therefore, each estimated effect is conditioned on age, i.e., the assessment of PFO-related and—unrelated factors is valid for patients of the same age who are consequently at the same time-dependent stroke risk. The multivariable model was pre-specified to avoid bias and an increased risk of data-driven false-positive findings (21).

A nomogram was developed to illustrate the effect size of factors. Hypothesis testing was performed on exploratory two-sided 5% significance levels.

Of note, our main research goal was identification and effect estimation of potential risk factors rather than hypothesis testing. Moreover, the current study design did not allow for sample size calculation and thus might not have been adequately powered to test the multiple null hypotheses that the respective regression coefficients are zero.

All analyses were performed using the statistical software R 3.6.1 (The R Foundation for Statistical Computing, Vienna, Austria).

## Results

### Factors Associated With Prior Stroke

Patient baseline characteristics are shown in Table 1. CS patients with prior stroke were significantly older (64.8 ± 10.8 vs. 53.3 ± 14 years), showed a higher body-mass-index (BMI, 27.8 ± 4.9 vs. 25.7 ± 4.5), were more likely to suffer from hypertension (59 vs. 32%), diabetes mellitus (19 vs. 6%), hypercholesterolemia (72 vs. 49%), and coronary artery disease (11 vs. 5%) and had a higher portion of right-to-left shunt (RLS) at rest (43 vs. 28%) compared to those with first-ever stroke. Adjusting for age, the odds ratio for these factors in the univariable model was 1.07, 1.09, 2.93, 3.37, 2.67, 2.57, and 2.00 for RLS at rest, respectively (Table 2). As expected, patients with prior stroke were frequently on antithombotic (72 vs. first-ever stroke 12%), antihypertensive (51 vs. 23%) and lipid lowering drugs (49 vs. 10%; all *p* < 0.0001).

**Table 1:** Baseline demographic, clinical and imaging data (missing values were imputed).

	**First-ever stroke *n* = 635**	**Missing values**	**Prior stroke *n* = 53**	**Missing values**	***P*-value**
Age, years	53.3 ± 14.0	–	64.8 ± 10.8	–	<0.001
Male gender	262 (41.3)	–	20 (37.7)	–	0.722
Body mass index	25.7 ± 4.5	29	27.8 ± 4.9	2	0.003
Hypertension	206 (32.4)	1	31 (58.5)	–	<0.001
Diabetes mellitus	41 (6.5)	2	10 (18.9)	–	0.003
Hypercholesterolemia	309 (48.7)	20	38 (71.7)	2	0.002
Smoking	168 (26.5)	16	11 (20.8)	2	0.456
Migraine	157 (24.7)	34	11 (20.8)	4	0.631
Coronary artery disease	30 (4.7)	8	6 (11.3)	1	0.050
Aortic plaque	16 (2.5)	–	3 (5.7)	–	0.174
Valsalva maneuver	51 (8.0)	57	1 (1.9)	4	0.169
Deep vein thrombosis	27 (4.5)	31	4 (7.7)	1	0.298
Pulmonary embolism	14 (2.3)	28	1 (1.9)	1	1.000
Right–to–left shunt at rest	176 (27.7)	–	23 (43.4)	–	0.024
Atrial septal aneurysm	215 (33.9)	–	20 (37.7)	–	0.674
**Medication on admission**					
Antithrombotic therapy	77 (11.9)		38 (71.7)		<0.001
Antiplatelet	66		35		
Oral anticoagulation	11		3		
Antihypertensive drugs	145 (22.5)		27 (50.9)		<0.001
Lipid lowering drugs	65 (10.1)		26 (49.1)		<0.001

*Values are mean ± SD or n (%)*.

**Table 2:** Association of baseline characteristics with prior stroke–univariate analysis (missing values were imputed).

**Predictor variable**	**OR**	**95% CI**	***P*-value**
Age, years	1.07	1.05–1.10	<0.001
Male gender	0.86	0.48–1.52	0.617
Body-mass-index	1.09	1.03–1.14	0.002
Hypertension	2.93	1.67–5.25	<0.001
Diabetes mellitus	3.37	1.51–6.96	0.002
Hypercholesterolemia	2.67	1.47–5.10	0.002
Smoking	0.73	0.35–1.40	0.365
Migraine	0.80	0.38–1.53	0.519
Coronary artery disease	2.57	0.93–6.11	0.045
Aortic plaque	2.32	0.53–7.26	0.192
Valsalva maneuver	0.22	0.01–1.04	0.138
Deep vein thrombosis	1.77	0.51–4.74	0.304
Pulmonary embolism	0.85	0.05–4.37	0.879
Right-to-left shunt at rest	2.00	1.12–3.53	0.017
Atrial septal aneurysm	1.18	0.65–2.10	0.568

*OR, odds ratio; CI, confidence interval*.

The pre-specified multivariable logistic regression (Table 3) demonstrated that prior stroke was strongly associated with advancing age (OR 1.06, 95%CI 1.04–1.10, *p* < 0.001), RLS at rest (OR 1.88, 95%CI 1.00–3.47, *p* = 0.046), hypercholesterolemia (OR 1.90, 95%CI 1.00–3.73, *p* = 0.055) and BMI (OR 1.06, 95%CI 0.99–1.13, *p* = 0.074), reaching statistical significance for age and RLS at rest. Moreover, the presence of a DVT (OR 1.76, 95%CI 0.46–5.44, *p* = 0.361) as well as an absent VM just before stroke onset (OR 0.28, 95%CI 0.02–1.39, *p* = 0.218) also hinted at a strong association with prior stroke, but was not statistically significant in this cross-sectional analysis.

**Table 3:** Association of baseline characteristics with prior stroke–multivariable analysis (pre-specified, missing values were imputed).

**Predictor variable**	**OR**	**95% CI**	***P*-value**
Age, years	1.06	1.04–1.10	<0.001
Male gender	0.78	0.41–1.46	0.446
Body-mass-index	1.06	0.99–1.13	0.074
Hypertension	1.06	0.53–2.11	0.872
Diabetes mellitus	1.45	0.58–3.42	0.413
Hypercholesterolemia	1.90	1.00–3.73	0.055
Smoking	1.35	0.61–2.82	0.439
Valsalva maneuver	0.28	0.02–1.39	0.218
Deep vein thrombosis	1.76	0.46–5.44	0.361
Right-to-left shunt at rest	1.88	1.00–3.47	0.046
Atrial septal aneurysm	0.98	0.51–1.84	0.959

*OR, odds ratio; CI, confidence interval*.

**Figure 1:**
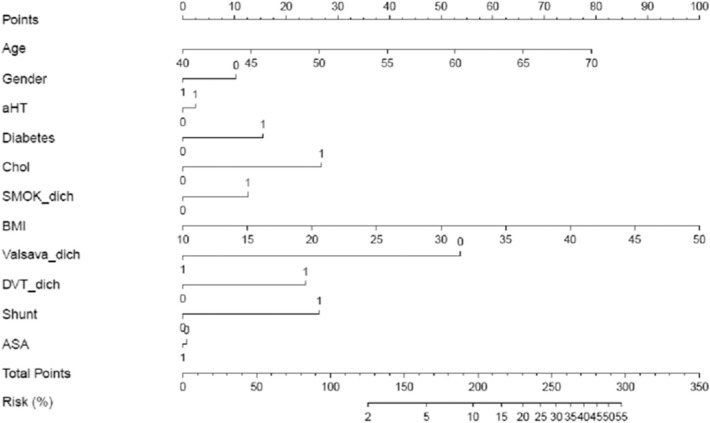
Nomogram: Likelihood of prior stroke in patients with cryptogenic stroke and PFO. Draw a line up perpendicular from the corresponding axis of each predictor variable to the top line labeled “points.” Sum up the number of points for all predictor variables to receive “total points. Now, draw a line descending from the “Total Points” axis until it intercepts the “Risk (%)” axis to estimate the likelihood of prior stroke.

Considering the weight of each predictor variable in the pre-specified multivariable model, reflected by its Odds Ratio, we developed a nomogram to illustrate the strength of each relation to prior stroke (Figure 1). Accordingly, age, BMI, hypercholesterolemia, RLS at rest, absence of VM directly preceding stroke onset and the presence of a DVT are the main factors associated with stroke recurrence.

For example, a 55-year-old (+40points) female (+10p) CS patient with PFO and an RLS at rest (+27.5p), BMI 30 kg/m^2^ (+50p), presence of VM just before stroke onset (0p), sonographic proof of ASA (0p) and DVT (+25p), known hypercholesterolemia (+27.5p), no arterial hypertension (0p) or diabetes (0p) and non-smoker (0p) sums up to a total of 180 points, which corresponds to a likelihood of 7–8% that this women belongs to the patient group with prior stroke.

## Discussion

The present analysis revealed associations of prior stroke with both PFO-related and -unrelated risk factors. Our study gives a novel insight into the nature and strength of the relationship of previous strokes at presentation and PFO.

Previous clinical and/or radiological stroke at presentation has been associated with higher risk of stroke recurrence in some studies (18) but not in others (22). In addition, recent data suggest that only CS patients with PFO in the high Risk of Paradoxical Embolism (RoPE)- Score strata, i.e., absence of classical vascular risk factors such as hypertension, diabetes mellitus and advancing age show an association of prior stroke with stroke recurrence (15). Age might play a dual role in the pathogenesis of stroke recurrence—both as a PFO-unrelated and PFO-related factor. It is usually considered a stroke risk factor that operates through PFO-unrelated pathogenic mechanisms. The increasing prevalence of classical vascular risk factors in older patients and the fact that stroke recurrence after PFO closure was higher in patients > 55 years of age than in younger patients underlines the relevance of PFO-unrelated contributors to stroke recurrence (23, 24). On the other hand, age might also increase the PFO-related stroke risk by prolonging the exposure time to Right-to-Left-Shunt. Prothrombotic conditions like endothelial damage, hypercoagulability, chronic inflammation, and venous stasis due to decreased regular exercise, which may not be addressed during routine stroke workup or may even be undetectable, accumulate with age and can predispose to paradoxical embolism in the long term (25).

The association of PFO-related factors with stroke recurrence has never been reliably established. Large PFOs have been positively associated with stroke recurrence in some studies (26–28) but not in others (14, 15, 29–31). The recent CLOSE and DEFENSE trials (7, 9) enrolled carefully selected cryptogenic stroke patients with large PFOs or concomitant atrial septal aneurysm. The studies showed that PFO closure was more efficacious in reducing the risk of stroke recurrence than antithrombotic treatment alone. The GORE-REDUCE trial included predominantly patients with moderate to large RLS and likewise demonstrated the superiority of PFO closure over medical treatment alone in preventing recurrent stroke (8). In addition, recent meta-analyses of RCTs comparing percutaneous PFO device closure with medical therapy in CS patients further support device closure in patients with certain PFO characteristics in particular moderate to large shunts (32, 33). Since PFO closure cannot prevent strokes of other possible etiologies, the findings of the above studies further emphasize the role of PFO-related factors in the pathogenesis of stroke recurrence.

Although our data suggest a strong association of prior stroke with conditions predisposing to paradoxical embolism such as DVT (OR 1.76) and VM directly preceding stroke onset (OR 0.28), the evidence is currently weak (DVT *p* = 0.361, VM *p* = 0.218) and needs confirmation in prospective, adequately powered trials. Briefly, the prevalence of DVT in the lower extremities, which was systematically captured in our database, was 4.4% in patients with first-ever stroke and 7.6% in patients with prior stroke. The findings are in keeping with the results of previous studies (34). However, we did not assess the prevalence of pelvic vein thrombosis in all patients. Paradoxical emboli originating from the pelvis have been recognized as an alternate source of stroke in this population (35). The missing data on pelvic vein thrombosis as well as the cross sectional study design may have obfuscated a statistical significant association between DVT and prior stroke.

VM at stroke onset was associated with a 72% reduced likelihood of a previous ischemic event. This could be best explained by the fact that VM increases RLS volume and supports a causal relationship between stroke and PFO, i.e. the stroke is most likely attributable to the PFO. PFO attributable strokes in turn demonstrated a low recurrence rate (36).

In terms of PFO-unrelated factors, our study identified hypercholesterolemia (OR 1.90, *p* = 0.055) and higher BMI (OR 1.06, *p* = 0.074) as being strongly associated with prior stroke, albeit not adequately powered to demonstrate statistical significance. Hyperlipidemia, especially an elevated ratio of ApoE/A1 or non-HDL/HDL levels, are known risk factors for ischemic stroke (37). Lipid-lowering drugs are firmly established in secondary stroke prevention (38). Just recently, it was shown that lowering LDL-levels below 1.8 mmol/l after stroke/TIA reduces the risk of a subsequent cardiovascular event compared to higher target LDL-levels (39), and the new ESC-guidelines recommend even lower LDL-levels in selected high-risk patients (40).

Several observational studies point to a lower rate of stroke recurrence in overweight or obese patients (41–44). However, recent studies in stroke patients receiving intravenous thrombolysis or patients with mild symptoms did not detect this relationship, thus challenging the “obesity paradox” (45, 46). Obesity was more common among patients with multiple CS and PFO in a single study, though the recurrence risk was not independently associated with BMI (18). Given these controversial findings, the impact of BMI on stroke recurrence needs further elucidation. Particularly in CS patients with PFO, elevated BMI and the presence of obstructive sleep apnea (OSA) might play a relevant role. Just recently, the coexistence of PFO and OSA in overweight men was suggested as a risk factor for wake-up stroke (47). Moreover, prolonged OSA episodes promoted RLS occurrence during sleep, which might increase the exposure time for paradoxical embolism (48).

The present study is limited by the missing assessment of OSA and other potentially high-risk PFO characteristics such as the presence of an Eustachian valve, a Chiari network or left atrial enlargement (49). In addition, the International PFO consortium study did not collect data on history of migraine stratified into those with aura or without. Furthermore, in patients with prior stroke, the PFO features were assessed at the time of the recurrent stroke only (i.e., at study enrollment). However, it is very unlikely that shunt size or presence of ASA would have changed substantially over time. Third, the process of screening for PFO across the 19 participating stroke centers was not standardized and thus might differ. Fourth, the effect of age cannot be separated from the time-dependent stroke risk. Therefore, age was mainly considered a confounder to allow adjusted effect estimation of other risk factors considered in the models.

Finally, we developed a nomogram to better illustrate the effect size of each risk factor and to easily estimate the probability of having suffered from a prior ischemic event at the time of the index stroke. Several studies suggest that prior stroke might also be associated with stroke recurrence (50, 51). Due to the cross sectional design of our study, we are currently not able to firmly establish those factors as risk factors for future events. However, the present study allows to identify promising “risk factor” candidates for recurrent stroke to be then tested in a longitudinal study design.

## Conclusion

In CS patients with PFO, RLS at rest, hypercholesterolemia and higher BMI were strongly related to prior stroke. The likelihood of prior stroke is associated with both, PFO-related and -unrelated factors. Based on the present findings, the impact of these factors on stroke recurrence in CS patients with PFO need to be further established in a longitudinal study design now.

## Data Availability Statement

Data that is not available with the article will be provided in an anonymized form by the corresponding author upon reasonable request from any qualified investigator (subject to the provisions of the IRB).

## Ethics Statement

The studies involving human participants were reviewed and approved by Ethikkommission Nordwest- und Zentralschweiz (EKNZ 2014-031). The patients/participants provided their written informed consent to participate in this study.

## Author Contributions

TK, M-LM, and KN contributed conception and design of the study. AH performed the final statistical analysis. TK and KN drafted the manuscript and all authors critically revised it for important intellectual content. All authors substantially contributed to the acquisition, analysis or interpretation of data and approved the final version of the manuscript.

## Conflict of Interest

BM and KN received speaker's fees from Abbott. DT is a member of the RESPECT trial steering committee and received fees from Abbott. The remaining authors declare that the research was conducted in the absence of any commercial or financial relationships that could be construed as a potential conflict of interest.
